# The use of fasting vs. non-fasting triglyceride concentration for estimating the prevalence of high LDL-cholesterol and metabolic syndrome in population surveys

**DOI:** 10.1186/1471-2288-11-63

**Published:** 2011-05-10

**Authors:** Jouko Sundvall, Jaana Leiviskä, Tiina Laatikainen, Markku Peltonen, Veikko Salomaa, Mauno Vanhala, Eeva Korpi-Hyövälti, Jukka Lauronen, Georg Alfthan

**Affiliations:** 1 National Institute for Health and Welfare, Department of Chronic Disease Prevention, Helsinki, Finland; 2 Central Finland Central Hospital, Unit of Family Practice, Jyväskylä, Finland and University of Eastern Finland, Department of Public Health and Clinical Nutrition, Kuopio, Finland; 3 Seinäjoki Central Hospital, Department of Internal Medicine, Seinäjoki, Finland

## Abstract

**Background:**

For practical reasons it is not easy to obtain fasting samples in large population health surveys. Non-fasting triglyceride (Tg) values are difficult to interpret. The authors compared the accuracy of statistically corrected non-fasting Tg values with true fasting values and estimated the misclassification of subjects with high low-density lipoprotein cholesterol (LDL-C) and the metabolic syndrome.

**Methods:**

Non-fasting blood was obtained from a population-based sample of 4282 individuals aged 24-75 years in the National FINRISK 2007 Study. Fasting blood samples were drawn from the same persons 3 months later. Non-fasting serum Tg values were converted into fasting values using previously published formula. LDL-C was calculated and classification of the metabolic syndrome was carried out according to three different latest guidelines.

**Results:**

The median (25^th^, 75th percentile) non-fasting serum Tg concentration was 1.18 (0.87, 1.72) mmol/L and after postprandial correction 1.06 (0.78, 1.52) mmol/L. The true-fasting serum Tg concentration was 1.00 (0.75, 1.38) mmol/L (*P *< 0.001) vs. non-fasting and corrected value. Bias of the corrected value was +5.9% compared with the true-fasting Tg. Of the true fasting subjects, 56.4% had LDL-C ≥3.00 mmol/L. When calculated using non-fasting serum Tg, the prevalence of high LDL-C was 51.3% and using statistically corrected Tg it was 54.8%. The prevalence of metabolic syndrome was 35.5% among fully fasted persons and among non-fasting subjects 39.7%, which after statistical correction of Tg decreased to 37.6% (P < 0.001 for all comparisons).

**Conclusions:**

Correction of non-fasting serum Tg to fasting values plays a minor role in population studies but nevertheless reduces misclassification of calculated high LDL-C from 5.1 to 1.6% and the metabolic syndrome from 4.2 to 2.1%.

## Background

For practical reasons fasting blood samples are difficult to obtain in large population studies and thus such studies commonly comprise non-fasting or 'semi-fasting' blood samples [1-5]. For example, in a long series of population-based studies in Finland since the early 1970s, a four hour fasting has been requested. Fasting is not a necessary prerequisite for many analytes, but for serum triglyceride (Tg) it is important [2,6]. Fasting serum Tg values are needed for cardiovascular disease risk evaluation [7,8] for the calculation of low-density lipoprotein cholesterol (LDL-C) concentration and for the classification of subjects into those with and without metabolic syndrome [9-11]. However, non-fasting Tg has emerged recently as an increasingly important risk factor [12,13]. Also estimating trends in these risk factors has become of utmost importance in epidemiology [14].

Recently, we derived correction factors whereby insufficiently fasted/postprandial serum Tg values can be converted into "corrected" fasting values [15]. In view of the potential error resulting from using non-fasting serum Tg values in the calculation of LDL-C and consequently in the classification of subjects into the metabolic syndrome, we transformed non-fasting data to fasting data using our recently published factors [15].

The aim of the present study was to assess how much a corrected non-fasting serum Tg deviates from the true fasting value and whether the correction for non-fasting serum Tg is worthwhile for calculation of LDL-C or for classifying subjects into the metabolic syndrome in health examination surveys.

## Methods

The National FINRISK 2007 Study (FR07) is the eight consecutive population-based risk factor survey in Finland. The surveys have been carried out with 5-year intervals since 1972 and the sample sizes have varied from 6500 to 13 500 men and women, depending on the survey year. The total sample size of the FR07 study was 9957 persons in the age range of 25-74 years. It was a random sample, drawn from the population register and stratified according to sex, 10-year age group and five geographical areas. Of the 9957 invited persons, 6247 (62.7%) took part in both the health examination and blood sampling [3].

During the year 2007 the same subjects participated in health examinations twice. In January-April, subjects were requested to fast for at least 4 hours before phlebotomy. The participants of the surveys were asked the time in full hours since their last meal by a study nurse. For the present analysis (non-fasting FR07, visit 1) eligible were those who reported having fasted ≥2 to ≤8 hours (1979 men) and ≥2 to ≤7 hours (2303 women) based on our earlier work on effects of fasting on Tg values [15]. In the substudy carried out in May-June the same participants were requested to fast for 10 hours (true-fasting FR07, visit 2).

In addition to the two groups described above, we identified a separate group drawn from the FR07 sample comprising 552 subjects who fulfilled the following fasting criteria: ≥8 hours for men and ≥7 hours for women in visit 1 and who had also participated in the true-fasting study, i.e., visit 2. These subjects are referred to as the Reference Group and they are not included in either of the above parent population samples. Individuals with serum Tg values >10 mmol/L were excluded from all analyses.

In addition to reporting the results on all men and women, we defined categories of healthy men (n = 824) and women (n = 994) as those who had a BMI ≤35; reported an alcohol consumption below the 90^th ^percentile in self-reported questionnaire; had no diagnosed cardiovascular disease (CVD), diabetes or cancer; had no medication for hypercholesterolemia; and had normal blood pressure. The results are also presented for subgroups with severe obesity BMI>35 (men n = 101, and women n = 183).

Subjects were classified into a high LDL-C group by using the cut-off value ≥3.00 mmol/L calculated by the Friedewald formula [16]. Subjects were classified as having the metabolic syndrome according to three different definitions [9-11].

### Collection of blood and biomarker analyses

All laboratory measurements were carried out at the Disease Risk Unit of the National Institute for Health and Welfare, Helsinki. The testing laboratory of the Disease Risk Unit (No. T077) is accredited by the Finnish Accreditation Service, FINAS. Serum total cholesterol, HDL-cholesterol and Tg concentrations were measured from both non-fasting and true fasting blood samples and plasma glucose concentrations were measured from true fasting blood samples only.

Venous blood samples were drawn in a sitting position with a light stasis into a tube containing clot activator (Vacuette, Greiner Bio-One, Frickenhausen, Germany) in visit 1 (non-fasting study) and (Venosafe, Terumo Europe, Leuven, Belgium) in visit 2 (true fasting study) for the lipid assays and into a fluoride-citrate tube (Venosafe) for the glucose assay. Serum and plasma were separated by centrifuging at 2200 g within 1 hour of collection for 10 minutes at room temperature. Consequently serum and plasma were aliquoted into bar-code-labelled tubes and stored locally at a minimum temperature of -20°C and transported frozen to the laboratory once a week and stored at -70°C for analyses. All measurements were performed within one month from the blood draw on a clinical chemistry analyzer, Architect c8000 (Abbott Laboratories, Abbott Park, IL, USA). Total cholesterol, HDL-cholesterol, Tg and glucose concentrations were determined enzymatically using commercial reagents from Abbott Laboratories.

For standardising the measurements, the laboratory has taken part in the Lipid Standardization Program organised by the Centers for Disease Control and Prevention (CDC), Atlanta, USA and External Quality Assessment Schemes organised by Labquality, Helsinki, Finland. During the course of the study comprising 6 months in 2007, the precision between series expressed as coefficient of variation (CV_a_) was less than 1.5% for all analytes except for HDL-cholesterol whose mean CV_a _was 2.3%. The mean (SD) systematic errors (bias) were 0.8% (0.5%) for total cholesterol, -0.6% (1.4%) for HDL-cholesterol, -1.1% (1.2%) for Tg and 0.0% (2.7%) for glucose.

### Statistical analyses

**Figure 1:**
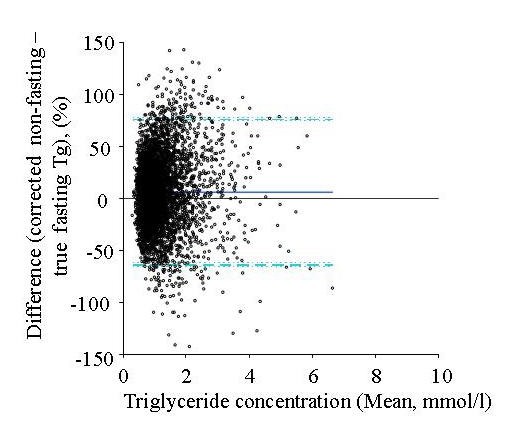
**Altman Bland plot of the differences between serum triglycerides of the non-fasting visit 1 with correction and the true fasting visit 2**. The lines describe identity (solid thin line), bias between results (solid thick line), 95% limits of agreement of bias (dashed line) and 95% confidence intervals of bias and limits of agreement (dotted lines).

The statistical analyses were done using Microsoft Excel Analyse-it software, Stata statistical package 10.1 (Stata-Corp. 2007. Stata Statistical Software: Release 10.1. College Station, TX; StataCorp LP.) and IBM SPSS Statistics 19. The bias comparisons were carried out using the Altman-Bland method [17] and shown as mean and 95% confidence intervals (95% CI), Figure 1. Serum Tg was expressed as mean, median and 25^th ^and 75^th ^percentiles. Significance for the differences between groups was analyzed using the Wilcoxon matched-pairs signed-rank test and differences between prevalences by chi-square test. We considered a *P *value less than 0.05 as statistically significant. The total intraindividual biological variation, CV^2^_tb _= CV^2^_anal _+ CV^2^_biol _[18] for Tg was calculated using Tg concentrations for the reference group from the two visits and for the whole group (n = 4282) from the non-fasting and true fasting visits.

Non-fasting serum Tg values were converted individually into corresponding fasting Tg values using the correction factors published previously [15]. The correction factors for the subgroups were per hour of fasting, 3.7% (all), 4.3% (healthy) and 6.5% (BMI>35). The correction was calculated until 8 hours for men and until 7 hours for women.

### Ethical issues and information protection

The population health surveys were approved by the Ethical Committee of the Hospital District of Helsinki and Uusimaa. All participants gave their written informed consent prior to participation in the study. The information protection rules in the National Institute for Health and Welfare (former National Public Health Institute to 2009) were followed throughout.

## Results

**Figure 2:**
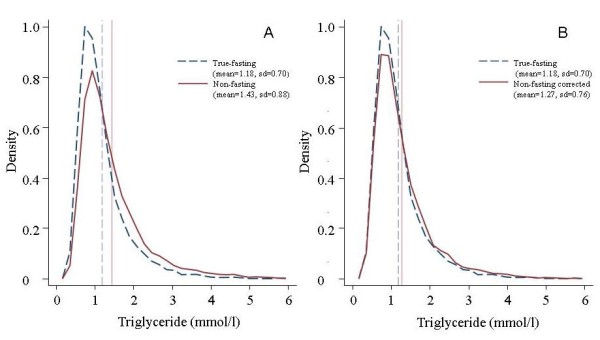
**Distribution of serum triglyceride results of the non-fasting visit 1, the true fasting visit 2 (panel A) and the corrected non-fasting visit 1 (panel B)**. The vertical dotted lines describe means.

Figure 1 shows the Altman-Bland plot indicating a 5.9% (95% CI: 4.8-7.0) bias between the difference of corrected FR07 and true-fasting data for serum Tg. Also, illustrated by the figure, the magnitude of the bias does not seem to depend on the Tg concentration. Figure 2 shows the distribution for raw data (all) (visit 1) before and after correction for non-fasting serum Tg concentrations of the subjects (n = 4282) and the true-fasting data (visit 2) of the same subjects.

The medians (25^th^, 75^th ^percentiles) of the non-fasting and corrected Tg data were 1.18 (0.87, 1.72) mmol/L (*P *< 0.0001) and 1.06 (0.78, 1.52) mmol/L (*P *< 0.0001) compared to the median of the true-fasting value, 1.00 (0.75, 1.38) mmol/L, Additional file 1. The median serum Tg concentrations of the non-fasting data differed significantly from true-fasting values for all groups, Additional file 1. After correction the corrected medians differed significantly from the true fasting medians for all subjects, all men, all women and healthy men, Additional file 1. The within sub-group difference between corrected and true-fasting median serum Tg was largest among all men 10.2% (*P *< 0.0001). In the reference group, in which all subjects had fasted for at least 7 hours at both visits (n = 552), the difference between the two visits was less than 2%, Additional file 2.

### Biological variation

The mean (25^th^, 75^th ^percentiles) total intraindividual biological variation (CV_tb_) of serum Tg of the reference group (n = 552) and the whole group (n = 4282) were 17.1% (6.5, 23.9) and 21.9% (8.3, 31.5), respectively. The corresponding mean CV_tb _values of serum total cholesterol were 6.1% (2.2, 8.1) and 6.2% (2.3, 8.4).

### Effect of triglyceride correction on the prevalence of high LDL-cholesterol

Among the 4282 subjects in the true-fasting sampling (visit 2), 56.4% had an LDL-C value ≥3.00 mmol/L. The proportion of high LDL-C in FR07 calculated using non-fasting (visit 1) serum Tg values was 51.3% and using statistically corrected serum Tg values 54.8% (P < 0.0001 for all comparisons). In the 7- or 8-hour fasting reference group, visit 1 (n = 552) the proportion of subjects with an LDL-C value ≥3.00 mmol/L was 58.5% and the respective value of the 10-hour fasting reference group, visit 2 was 57.1%.

### Effect of triglyceride correction on the prevalence of the metabolic syndrome

The metabolic syndrome (Tg ≥ 1.7 mmol/L) according to the National Cholesterol Education Program 2005 (MS-ATP) criteria [11] was found in the fully fasted visit 2 in 1918 subjects (35.5%). In the non-fasting subjects the proportion was 39.7%, which after correction for postprandial serum Tg decreased to 37.6% (difference between prevalences P < 0.0001). In the reference subgroup, which had fasted at both visits, the prevalences were 35.9% (198 subjects) in visit 1 and 36.4% (201 subjects) in visit 2.

Based on criteria of the International Diabetes Federation (MS-IDF) [10] and the International Diabetes Federation Task Force (MS-IDFTF) [9] the prevalences were 47.1% and 49.8% in visit 1, 44.8% and 46.3% in visit 2 and 45.6% and 47.9% after statistical correction of serum Tg in visit 1, respectively (P < 0.0001 for all respective comparisons). Misclassification due to use of non-fasting vs. corrected Tg concentrations in calculation of prevalences decreased from 4.2% (metabolic syndrome visit 2-visit 1) to 2.1% (visit 2-corrected visit 1) using the MS-ATP criteria. Likewise, misclassification using the MS-IDF or MS-IDFTF criteria improved from 2.3% to 1.5% and 2.5% to 1.9%, respectively.

## Discussion

In population health examination surveys standardization and long-term quality assessment of risk factor measurements are essential. Especially so in monitoring trends and tracking of risk factors [3,5,19]. Among the major risk factors for CVD, serum Tg has emerged recently as an increasingly important risk factor [7,12,13,20,21]. In both diagnostics and risk assessment fasting serum Tg concentration is required either for the calculation of LDL-C or classification of subjects into the metabolic syndrome. In large population-based health examination surveys true fasting for all subjects is often difficult to achieve [1-5,22] and usefulness of undefined post prandial serum Tg data is doubtful. In a previous study, we calculated factors by which non-fasting serum Tg values can be converted into fasting values when the time from the last meal is known [15].

In the present study we wanted to test whether the statistical correction of non-fasting serum Tg into fasting values is worthwhile in calculating LDL-C and categorizing subjects into the metabolic syndrome. Our data showed that using incomplete fasting serum Tg concentration to calculate LDL-C concentration results in misclassification of 5.1% of subjects as having an LDL-C below 3.00 mmol/L compared to true fasting values. Correction of post prandial serum Tg narrowed misclassification to 1.6%, increasing the number of subjects at risk by some tens of thousands at the population level in Finland. Likewise, categorizing subjects for the metabolic syndrome by the MS-ATP-2005 [11] criteria using corrected non-fasting Tg instead of the non-fasted values, decreased the proportion of misclassification and resulted in virtually the same prevalence as the use of true-fasting serum Tg, the difference being 2.1 percentage units. According to MS-IDF-2005 [10] and MS-IDFTF-2009 [9] criteria the differences were very similar.

The total intraindividual biological variation of fasting serum Tg in the reference group was 17.1%, which is much narrower than in other similar studies [23] but the total biological variation in our original samples (21.9%) was of the same order of magnitude as found by others, 21.7 - 29.9% [18], having a time frame of months between the phlebotomies. The small effects of non-fasting vs. fasting serum Tg on misclassification into high LDL-C or metabolic syndrome should be compared to the intraindividual biological variation of serum Tg. Because of the high biological variation of serum Tg several measurements of Tg per subject are needed for reliably estimating the CVD risk [7,23].

In addition to its use as a risk factor, serum Tg concentration is used widely to calculate LDL-C with the Friedewald formula [16]. Because of limitations of the formula [24], the new recommendation from the National Cholesterol Education Program (NCEP) encourages direct measurement of LDL-C. Increasing concerns have, however, been raised about the specificity of different direct methods for measuring the cholesterol concentration from various lipoprotein fractions. The newly published data showed [25] that all evaluated direct LDL-C methods failed to meet the NCEP's total error goals when analysing samples from patients with cardiovascular disease or dyslipidemia. Recent data have proved that the number of LDL particles is a more reliable indicator for cardiovascular risk assessment than the cholesterol concentration in lipoprotein particles [26]. Because there is only one apolipoprotein B (apoB) molecule per one LDL particle, measurement of apoB gives an estimate of the number of lipoprotein particles. Therefore, it has been suggested that, to improve CVD risk assessment, apoB measurement should be adopted in addition to LDL-C to national guidelines [27]. Implementation of such a measure would benefit greatly large surveys, since measurement of apoB does not require a fasting blood sample.

## Conclusions

Our data based on a random population sample of 4282 individuals examined twice within a 3 month interval showed that measuring serum Tg after an incomplete fast causes a relatively small misclassification of the prevalence of people with high LDL-C or metabolic syndrome.

## Competing interests

The authors declare that they have no competing interests.

## Authors' contributions

JS, JaL and GA designed together the study and drafted the manuscript; JaL supervised the laboratory analyses and JuL was responsible for data management and performed the statistical analyses; TL, MP and VS interpreted the cardiovascular data and MV and EKH were responsible for part of the data collection and contributed significantly to the final manuscript. All authors have read and approved the final version.

## Pre-publication history

The pre-publication history for this paper can be accessed here:

http://www.biomedcentral.com/1471-2288/11/63/prepub

## Supplementary Material

Additional file 1 **Triglyceride medians and means with and without correction among non-fasting subjects and among true fasting subjects**. Wilcoxon matched-pairs signed-ranks test values for the differences between the groups. Abbreviations: BMI, body mass index; FR07, FINRISK-2007 Study; P, probability. ^a^P-value without is comparison of non-fasting without correction with true fasting, with is comparison of non-fasting with correction with true fasting. Click here for file

Additional file 2 **Triglyceride medians and means, number and proportions of high LDL-C (>3.00 mmol/L) and high triglyceride (>1.70 mmol/L) concentrations in the different groups**. Abbreviations: FR07, FINRISK-2007 Study; LDL-C; Low-Density Lipoprotein Cholesterol; N, Number of Subjects; Tg, Triglyceride. ^a^P < 0.0001 compare to true fasting. Click here for file
