# Supplementary material for: The use of fasting vs. non-fasting triglyceride concentration for estimating the prevalence of high LDL-cholesterol and metabolic syndrome in population surveys
Source: BMC Med Res Methodol. 2011 May 10;11:63. doi: 10.1186/1471-2288-11-63 (PMC3112195; doi:10.1186/1471-2288-11-63)
Supplement: Additional file 1 — Triglyceride medians and means with and without correction among non-fasting subjects and among true fasting subjects. Wilcoxon matched-pairs signed-ranks test values for the differences between the groups. Abbreviations: BMI, body mass index; FR07, FINRISK-2007 Study; P, probability. aP-value without is comparison of non-fasting without correction with true fasting, with is comparison of non-fasting with correction with true fasting. [file 1471-2288-11-63-S1.DOC]

**Table 1: Triglyceride medians and means with and without correction among non-fasting subjects and among true fasting subjects.**

| Sample Group | No. of Samples | FR07 Non-fasting Without Correction | | FR07 Non-fasting With Correction | | FR07 True Fasting | | *P*-Value^a^  Correction | |
| --- | --- | --- | --- | --- | --- | --- | --- | --- | --- |
|  |  | Median | Mean | Median | Mean | Median | Mean | Without | With |
| All | 4282 | **1.18** | 1.43 | **1.06** | 1.27 | **1.00** | 1,18 | < 0.0001 | < 0.0001 |
| All men | 1979 | **1.36** | 1.64 | **1.18** | 1.43 | **1.07** | 1.30 | < 0.0001 | < 0.0001 |
| All women | 2303 | **1.06** | 1.26 | **0.96** | 1.14 | **0.95** | 1.08 | < 0.0001 | < 0.0001 |
| Healthy men | 824 | **1.19** | 1.47 | **1.03** | 1.25 | **0.98** | 1.18 | < 0.0001 | 0.0004 |
| Healthy women | 994 | **0.95** | 1.09 | **0.84** | 0.97 | **0.84** | 0.95 | < 0.0001 | 0.2743 |
| BMI>35 (men) | 101 | **2.02** | 2.34 | **1.55** | 1.83 | **1.54** | 1.78 | < 0.0001 | 0.3226 |
| BMI>35 (women) | 183 | **1.61** | 1.71 | **1.30** | 1.41 | **1.36** | 1.45 | < 0.0001 | 0.1392 |

Wilcoxon matched-pairs signed-ranks test values for the differences between the groups.

Abbreviations: BMI, body mass index; FR07, FINRISK-2007 Study; P, probability.

^a^P-value without is comparison of non-fasting without correction with true fasting; with is comparison of non-fasting with correction with true fasting.
