# Supplementary material for: The use of fasting vs. non-fasting triglyceride concentration for estimating the prevalence of high LDL-cholesterol and metabolic syndrome in population surveys
Source: BMC Med Res Methodol. 2011 May 10;11:63. doi: 10.1186/1471-2288-11-63 (PMC3112195; doi:10.1186/1471-2288-11-63)
Supplement: Additional file 2 — Triglyceride medians and means, number and proportions of high LDL-C (>3.00 mmol/L) and high triglyceride (>1.70 mmol/L) concentrations in the different groups. Abbreviations: FR07, FINRISK-2007 Study; LDL-C; Low-Density Lipoprotein Cholesterol; N, Number of Subjects; Tg, Triglyceride. aP < 0.0001 compare to true fasting. [file 1471-2288-11-63-S2.DOC]

**Additional file 2:** **Triglyceride medians and means, number and proportions of high LDL-C (**>**3.00 mmol/L) and high triglyceride (**>**1.70 mmol/L) concentrations in the different groups.**

| Group | No. of Samples | Tg mmol/L | | LDL-C ≥3.00 mmol/L | | Tg ≥ 1.70 mmol/L | |
| --- | --- | --- | --- | --- | --- | --- | --- |
|  |  | Median | Mean | N | % | N | % |
| FR07 non-fasting without correction | 4282 | 1.18^a^ | 1.43 | 2196 | 51.3 ^a^ | 1103 | 25.8 |
| FR07 non-fasting with correction | 4282 | 1.06 ^a^ | 1.27 | 2345 | 54.8 ^a^ | 822 | 19.2 |
| FR07 true fasting | 4282 | 1.00 | 1.18 | 2415 | 56.4 | 656 | 15.3 |
| Reference group, visit 1 | 552 | 1.03 | 1.22 | 323 | 58.5 | 88 | 15.9 |
| Reference group, visit 2 | 552 | 1.05 | 1.22 | 315 | 57.1 | 96 | 17.4 |

Abbreviations: FR07, FINRISK-2007 Study; LDL-C; Low-Density Lipoprotein Cholesterol; N, Number of Subjects; Tg, Triglyceride.

^a^P<0.0001 compare to true fasting
